# Fibroid Lung

**Published:** 1910-04-30

**Authors:** A. J. Jex-Blake


### Hospital Clinics.

### FIBROID LUNG.

By A. J. JEX-BLAKE, M.A., M.B., M.R.C.P.

(A Clinical Lecture delivered at the Brompton Hospital for Consumption.)

The first duty of anyone using the term "fibroid lung" is to make plain the sense in which he uses it; for during the last hundred years it has been employed in a great variety of senses by different authorities. There can be no doubt that nowadays it should bear only the sense given to it by Sir Andrew Clarke and Drs. Hadley and Chaplin in 1894—namely, that of a chronic disease of the lung, not tuberculous in origin, in which there is a diffuse fibrosis of more or less of the pulmonary tissue. It is important to insist on the fact that the tubercle bacillus has nothing to do with this form of fibrosis of the lung. Neglect of this distinction has introduced a great deal of confusion into the naming of the various fibrotic diseases of the lungs. Extensive fibroid transformation of pulmonary tissue is, of course, very common in chronic pulmonary tuberculosis; but the symptoms, course, and prognosis of this "tuberculo-fibroid disease" are so very different from those in "fibroid disease" of the lungs that the radical distinction between the two ought to be indicated in their names. For example, Bayle in 1810 described six cases of fibroid induration of the lung combined with melanosis; in 1865 Sutton went minutely into the morbid anatomy of "fibroid degeneration" of the lung; but in each of these instances it appears that the pulmonary lesions were really tuberculous in origin, although the fact is not indicated, as it should have been for the sake of clearness, in the naming of the pulmonary disease. Several other terms have been applied to the process resulting in the production of fibroid lung: Corrigan (1838) called it a "cirrhosis of the lung"; Sir Andrew Clarke (1868), "fibroid phthisis"; on the Continent "sclerosis of the lung" is that commonly used; but these names present no advantages, and, for the sake of uniformity, should be given up in favour of "fibroid disease of the lungs."

### Ætiology of Fibroid Lung.

The ætiology of fibroid disease of the lungs is not a little complex; it is always due to some previous pulmonary disorder, and never arises spontaneously. As Marfan says of it, "*ce n'est pas une maladie, c'est un aboutissant*"—it is not a disease by itself, but the legacy of some other complaint. It is not very common, although from its chronicity—one of my patients has had it now for 55 years—it must tend to appear less rare than it really is. The two sexes seem to be equally affected. It usually begins in youth or childhood; in 22 out of 38 cases collected at Guy's Hospital the onset was between the ages of 5 and 15, no doubt because in so many instances it follows after the broncho-pneumonia complicating whooping-cough and measles. Race and climate, occupation and hygiene, only influence the incidence of fibroid lung in so far as they affect the predisposition to diseases of the lung in general, and also in so far as they influence the ability of the patient to secure proper treatment and after-treatment for these diseases. Heredity, it need hardly be said, counts for little in the production of fibroid lung.

As regards its pathological anatomy, the situation of the fibrosis will naturally depend on the situation of the pulmonary lesion producing it. If this is a unilateral pleurisy with effusion, for example, the fibroid disease will affect the base of one lung. If it is a lobar pneumonia that has not resolved properly, the fibrosis will affect the corresponding lobe of the lung. When it is broncho-pneumonia that sets up the fibrotic process, then both lungs will be involved in the fibroid disease, as was the case in five out of Sir Andrew Clarke, Drs. Hadley and Chaplin's 45 patients. At the post-mortem the fibroid lung is commonly found to be much reduced in size, and very densely adherent to the parietal pleura over the fibroid area; often the pleura is found to be much thickened—up to ¾ inch. The fibroid part of the lung has a very tough, leathery consistency, squeaking or grating under the knife; its tissue is solid, sinking in water, and varies in colour from white or grey to black. More or less bronchiectasis is usually present, either cylindrical or saccular, and the vessels show thickening of their walls; the bronchial lymphatic glands are always enlarged, and more or less pigmented. In parts of the lung where the fibrosis is less complete the pulmonary tissue is seen to be traversed and cut up by hard, thickened fibrous band of a white or grey colour. In general, it may be said that the middle and base of the lungs are most affected, just as in pulmonary tuberculosis the most marked lesions are at the apices. The unaffected portions of the lungs show considerable compensatory emphysema. Microscopical sections of fibroid lungs show a diffuse fibrosis, the pulmonary tissue being more or less completely replaced by fibrous tissue. In the cases following lobar pneumonia, if they are examined within a few months of the onset of the acute disease, tufts of organised and vascular connective tissue may be found filling up the alveoli and taking origin from the alveolar walls. In the pleurogenous cases, on the other hand, attention should be directed to the lymphatics, which will be blocked or interrupted by inflammation within and around their walls.

### An Attempt at a Classification.

It is far from easy to give any comprehensive picture of the pathological anatomy of fibroid lung because the variety of the methods in which it may arise is so great. The earlier writers were content to give it a fairly simple pathogenesis; thus Charcot (1878) classified the "pulmonary scleroses" under three heads:—

(1) Pneumonic sclerosis, a rare but undoubted sequela of acute lobar pneumonia.

(2) Broncho-pneumonic sclerosis with bronchiectasis, secondary to broncho-pneumonia. This is the commonest mode of origin of fibroid lung, and was that noted in fourteen out of forty-five cases observed at Guy's Hospital.

(3) Pleurogenous sclerosis, due to pleurisy.

This is, of course, as much a pathological as a clinical classification. Later writers have added to the possible causes of fibroid disease of the lung, and Letulle (1890), after recapitulating those given above, adds three more:—

(4) Syphilitic sclerosis, a rare form producing the "poumon ficelé," or the "corded lung," of French authors.

(5) Traumatic sclerosis, as seen in the pneumoconioses.

(6) The toxic scleroses. Letulle takes an extremely wide view of the possibility that the toxins of all sorts of chronic diseases may produce fibroid lung, but admits that a toxic sclerosis has nothing characteristic about it, and is generally obscured by the local inflammatory condition of the lung (bronchitis, pneumonia, etc.) that is always associated with it.

But by far the most elaborate study of the pathogenesis of fibroid lung is that made by Sir Andrew Clarke and Drs. Hadley and Chaplin in 1894. They distinguish eleven possible causes for its production; the only toxic sclerosis they consider is that due to alcoholism, which, they believe, may sometimes be a cause of fibroid disease of the lung. To the six possible causes already given they add—

(7) Prolonged chronic bronchitis; which probably acts by blocking the lymphatic channels round the bronchi.

(8) Collapse of the lung, such as that due to plugs of mucus in the bronchi, or to occlusion of bronchi by the pressure of aneurysms or new growths.

(9) Bronchiectasis; but this may well be an effect of the fibroid disease, and not its cause.

(10) Trauma. This appears to have been the cause of the fibrosis in the case of one of my patients.

(11) The "Fibroid diathesis." This was strongly urged as a cause of pulmonary fibrosis by Handfield-Jones (1854); but the existence of any such diathesis seems to be highly doubtful.

Pathologically, fibroid lungs may be classified more simply, according as the origin of the fibrosis is mainly—

(*a*) alveolar;
(*b*) bronchial or broncho-pneumonic; or
(*c*) interstitial, lymphatic in the case of pleurogenous sclerosis, venous in the case of the fibrosis secondary to cardiac disease and chronic venous engorgement.

To sum the matter up, it may be said that the great majority of fibroid lungs arise after either broncho-pneumonia, lobar pneumonia, or pleurisy: in only a comparatively few cases is the condition due to any of the other eight causes enumerated. And in a few instances fibroid disease of the lung may be found when nothing in the previous history of the patient can be discovered to account for its occurrence.

### A Note on Symptomatology.

The symptoms of fibroid lung are very different in different cases—one writer goes so far as to say that no two cases are alike—and they also vary considerably in each single case according to stage of the disease. In the first place it may be said that the onset is acute, and very unlike the insidious onset characteristic of pulmonary tuberculosis. Most cases of fibroid lung begin with an acute attack of pneumonia, broncho-pneumonia, or pleurisy; and the recovery from these acute diseases is incomplete, and far slower than it is when the normal *restitutio ad integrum* takes place. The transition from the acute disease to the chronic fibroid state takes many months, during which the patient will be invalided and unfit for work, or, if he is a child, for school, although able to get about and to look after himself.

The three main symptoms throughout the disease are cough, expectoration, and dyspnœa. In the earlier stages, when the subacute inflammatory process commonly responsible for the fibrosis is still going on, and has not settled down to the quiescence implied in the term "fibrosis," the dyspnœa will be much more marked than it is in the later stages, and may be accompanied by cyanosis. In addition, the patient is likely to suffer from other symptoms, such as rapid pulse, palpitations of the heart on slight exertion, and more or less irregular low fever; anæmia and wasting are likely to be marked, and complaint of pain in the side on exertion or coughing will be made. After a few months, however, as the morbid process in the affected lung settles down, a gradual remission of these symptoms will be observed; the dyspnœa will lessen in proportion as cardiac compensation and readjustment of the pulmonary conditions take place, and a corresponding general improvement in the patient's health will be observed. The cough and expectoration will continue to some extent, however; and the extent will depend largely on the amount of bronchiectasis associated with the fibrosis. If there is little bronchiectasis the cough may be almost a negligible factor.

### Some Points in Diagnosis.

When the fibrosis has been established for some years it is surprising to see how little it may interfere with the customary avocations of the patients. They may be able to follow fairly laborious occupations without much dyspnœa or discomfort; their pulse-rate may be quite normal, and herein lies perhaps the most marked of the distinctions between fibroid disease of the lung and tuberculo-fibroid disease of the lung—that is to say, tuberculosis of the lung, in which there is extensive pulmonary fibrosis. In this, rapidity and irritability of the pulse are almost always present. If there is much bronchiectasis associated with the fibroid lung, then the cough and expectoration are likely to be no little trouble to the patient, and the septic processes that so often go on in his dilated bronchi will make him liable to feverish attacks and the symptoms that go with them. But in such patients the bronchiectasis is the main trouble, and the diagnosis should be "bronchiectasis with fibroid lung." Hæmoptysis may occur in these patients, just as in others epistaxis may come on after prolonged bouts of coughing. In advanced cases of many years' standing, in which old age with its senile decay, or intercurrent disease, occur to weaken the patient, other symptoms will arise that are no doubt in part due to the fibrosis of the lung. Wasting is likely to occur, with general debility in consequence; gastro-intestinal disturbances, diarrhœa—particularly if there is much bronchiectasis and expectoration—or albuminuria.

The physical signs of fibroid lung are always extensive, but they are not characteristic; that is to say, they may be identical with those of fibrotic pulmonary tuberculosis; it is in the course and duration of these two conditions that differences between the two must be looked for. During the establishment of the fibroid condition the signs, of course, vary considerably, according to the nature of the pulmonary disease that is producing it—broncho-pneumonia, lobar pneumonia, pleurisy, or whatever it may be.

### Physical Signs.

When the fibroid condition is well established, however, the physical signs will present a greater uniformity. On inspection, shrinkage and deformity of the affected side or over the affected parts of the lungs will be conspicuous, with a greater or less degree of limitation of movement on respiration. The obliquity and relative positions of the ribs will be altered, and on inspection of the back scoliosis will be generally observed. The concavity of this scoliosis will generally be towards the affected side in the dorsal region; if the curvature is marked, compensatory curves will be seen in the upper dorsal and cervical, and in the lower dorsal and lumbar regions. The shoulder will be seen to be held at a lower level on the affected side than on the other. The flattening of the chest wall will usually be less marked at the apex of the lung in fibroid disease; in fibrotic forms of pulmonary tuberculosis, on the other hand, the flattening is generally most developed at the apex. Examination of the chest by the *x*-rays will often show great limitation of movement of the diaphragm on the affected side, while the fibroid part of the lung is more or less opaque, with dark strands or wisps, corresponding to fibrous bands, passing out into the more healthy and transparent parts of the lung tissue near by.

On palpation the vocal fremitus is increased over the affected part of the lung and in its neighbourhood. Occasionally the fremitus of coarse bronchial rhonchi, possibly, too, of coarse pleural friction, can be felt. In many instances the shrinkage of the lung causes displacement of the heart towards the affected side, so that the apex-beat may be found in an abnormal situation, and the area of cardiac pulsation may be unusually extensive. Of course such displacement of the heart is best seen by the use of the *x*-rays.

On percussion, the fibroid part of the lung will be dull; the rest of the lung, and also the other lung, will be hyper-resonant, from the compensatory emphysema that endeavours to make good the shrinkage of the fibroid tissue.

On auscultation the signs are very variable in different cases. So much depends upon the position and extent of the fibrosis, and so much on the degree to which the bronchi are involved. If the base of one lung is affected, and there is little bronchiectasis, as often happens when the fibrosis follows pleurisy, the breath-sounds will be bronchial or tubular over the fibroid area, harsh in its immediate vicinity, and bronchophony will be heard. If there is much chronic bronchitis, or bronchiectasis is a feature of the case, all sorts of adventitious sounds, many of a consonating, clicking, or metallic character, will be heard. Often peculiar smacking or sucking sounds are present.

As regards the other organs and parts of the body, fibroid lung produces few physical signs apart from those in the lungs and thorax. Displacement of the heart is common; it should be particularly noted that disturbances of the heart's action are rare; the pulse-rate is normal, and it does not become excessively frequent on exertion unless the fibrosis is very extensive or recent. Clubbing of the finger and toe-tips is common, particularly in the bronchiectatic cases. In considering the complications of fibroid lung, it is not easy to say where the phenomena that may be regarded as normal to it end, and where the complications begin. Having a more or less extensive mass of fibrotic and highly unhealthy lung-tissue within them, the patients are more prone than normal persons to catch all sorts of pulmonary diseases, particularly bronchitis and tuberculosis. Hæmoptysis is not very uncommon, and may be very severe, as in bronchiectasis. Cardiac complications may occur towards the end of the disease, from over-taxing and failure of the right heart.

### Prognosis and Course.

The course and duration of fibroid lung are very variable, and have been very differently estimated by different writers. It is clear that much must depend on the extent of the pulmonary tissue involved; if this is very great, much strain will be put upon the right side of the heart; and the liability to fatal complications, such as bronchitis, pneumonia, gangrene of the lung, severe hæmoptysis, will be increased if there is much bronchiectasis. On the other hand, the duration is likely to be much longer if the fibrosis is limited, and if the patient is young, and therefore able to adapt himself better, as he grows up, to the abnormal pulmonary conditions. The average duration of the Guy's Hospital series of cases was 12 years, and many of these patients were still living at the time when the statistics were worked out. In three cases that I have under observation the durations have been 22, 39, and 55 years, and the first two of these patients are still able to work and earn their livings, while the third was so until three years ago.

The diagnosis of fibroid lung is far from easy in its earlier stages, though not so difficult when the condition has persisted for many years. It can never be made from the physical signs and symptoms alone. In the earlier stages, while the fibrosis is in process of becoming complete and the patient is still debilitated and barely free from slight feverish attacks or dyspnœa, the resemblance to pulmonary tuberculosis will be close, particularly if the fibrosis affects one of the apices. The diagnosis between the two can only be made when repeated examinations of the sputa have shown that tubercle bacilli are constantly absent, and when the lapse of time has proved the tendency towards improvement and recovery. The various tuberculin reactions—Calmette's, von Pirquet's, or Moro's—might be of considerable assistance here. A chronic pleural effusion, whether serous or purulent, might be very difficult to diagnose from an early fibroid lung, particularly if it were not situated at the base of the pleural cavity, but in between two lobes of the lung; puncture with an aspirating needle, and probably repeated puncture, would be the best means of discriminating between the two conditions. I have seen a case of what turned out to be invasion of the base of the right lung by an endothelioma of the scapula, associated with an acute pneumonia, in which extensive consolidation at the right base was left after the acute disease was over. Chronic pleural effusion or empyema was diagnosed, and the base of the right lung was punctured repeatedly without success, a general anæsthetic being given on the second occasion when puncture was attempted. Some months later the patient attended at another hospital, and from the physical signs and history the diagnosis of fibroid lung after lobar pneumonia was made, with malignant disease of the scapula. At the post-mortem, about a year later, however, it was found that the lung was not fibrotic, but that its base was pushed up and replaced by a mass of new growth, an endothelioma, spreading in from the scapula; and it appears probable that it was this mass that was diagnosed first as a pleural effusion and later as a fibroid lung. Hence it is possible that mediastinal new growths, whether primary or secondary, may simulate fibroid lung so far as the physical signs go; the downward progress of the case would give the diagnosis, being rapid with the new growth, extremely slow with the fibroid lung.

The prognosis, once the diagnosis of fibroid lung is thus established, is good. The patient may fairly look forward to many years of useful life in most instances; the presence of very extensive fibrosis, or of much bronchiectasis, or of conditions of life in which the patient can take no sort of care of himself, all make the prognosis in any particular instance less favourable.

### Prophylaxis and Treatment.

I think there is no doubt that something might be done in the way of prophylaxis against the development of fibroid lung. The majority of cases arise from either broncho-pneumonia, lobar pneumonia, or pleurisy; and the careful treatment, and particularly the careful after-treatment of these acute diseases, with a view to procuring their thorough cure and to the prevention of their lapsing into a sub-acute or chronic condition, would do much to lessen the frequency with which fibroid lung occurs. The patients should be kept at rest until resolution of the acute pulmonary disease has occurred, and, in order to promote the absorption of the pathological exudates in or about the lung, should not be allowed to get about or go to work until they are really fit.

The treatment of fibroid disease of the lung must be on general lines, and symptomatic. The patient should conduct his life in accordance with the ordinary rules of hygiene, taking particular care to avoid anything that might irritate the lungs or cause bronchitis. This is particularly the case with those patients who have much bronchiectasis associated with the fibrosis. The patients should be encouraged to lead an active life, within limits that they will be able to find out for themselves. I am not aware of any drug that will directly benefit the fibrotic state of the lungs, once it has been established; numerous authors have tried subcutaneous injections of fibrolysin in patients with slowly resolving lobar pneumonia, thickened pleura, or obstinate pleural effusion, and have been satisfied that good resulted from the treatment. This, however, is preventive rather than curative, so far as regards fibroid lung. A drug that has been strongly recommended by several French writers is benzoate of sodium, in doses of from 30 to 150 grains a day, but I have no experience of its use myself. The chief symptoms for which patients with fibroid disease of the lungs will seek relief are the cough, which is often paroxysmal, and the expectoration, which will often have a sickly nauseous odour or may even be fœtid. Treatment for these should be on the ordinary lines.

The following are brief notes of three cases of fibroid lung now under observation:—

### Cases.

1. E. T., male, aged 55, electrician. At 16 he had a severe attack of pleurisy on the right side, which laid him up for six months. Since then he has been troubled on and off with cough and expectoration, the latter never fœtid: this has never been severe enough to lay him up for more than a few days at a time. In the last 10 years he has had numerous attacks of articular gout. He is a well-nourished, muscular, healthy-looking man. His left chest shows considerable flattening and shrinkage, being 1 to 2 inches less in circumference than his right chest. The dorsal spine shows scoliosis, the concavity being to the left. There is evidence of much diffuse fibrosis of the left lung, mainly at the base. The heart is displaced to the left. There is little evidence of bronchiectasis. The sputum has been repeatedly examined for tubercle bacilli during the last four years, always with a negative result. The fingers are slightly clubbed.

2. E. O., female, 48, housemaid. In July 1887 she had pleurisy with effusion at the right base, and was seen at the Brompton Hospital for Consumption in October 1887 by Dr. Kingston Fowler, to whose courtesy I am indebted for permission to make use of the notes he then took of her case. He found that the base of the right lung was collapsed and dull on percussion, and that the chest-wall over it was retracted. For the last 22 years she has been at work, with fairly frequent short breakdowns in health from "cough." She now is a pale, thin woman, fully looking her years. The right side of her chest is 1½ to 2 inches smaller than the left, and moves ill on respiration; the right shoulder is carried lower than the left. The right lung generally is duller on percussion than the left, and this is particularly the case at the base. The heart-sounds are normal, and the heart is not displaced. Dorsal scoliosis is present, convex to the right. Examination with the $x$-rays shows that the outer or lower parts of the right lung are abnormally opaque, and that the vertical excursions of the right half of the diaphragm are only half those on the left. The finger-tips are not clubbed. Tubercle bacilli have not been found in the sputum.

3. G.C., male, aged 67, carpenter, unable to work for three years. At the age of twelve he fell from a height of 80 feet, injuring his right chest. A few months later his mother noticed that his right chest had shrunk. Till he was 45 he had little trouble; since then he has had a cough on and off. In the last two years he has been laid up for three months in an infirmary; the cough has been bad for three years. He is a thin, emphysematous but fairly healthy-looking man. There is marked cyphosis. The right chest is smaller than the left, and moves ill on respiration. It is generally dull on percussion. Seen by the $x$-rays it shows no very definite abnormal shadows. The fingers are not clubbed, and the heart is not displaced.

